# Design of a porous biodegradable internal fixation for the treatment of pauwels type III femoral neck fractures

**DOI:** 10.3389/fbioe.2025.1639459

**Published:** 2025-10-31

**Authors:** Chunsheng Liu, Songyuan Wang, Yinuo Zhao, Ying Shen, Meng Zhang, Haoqian Li, Yanqin Wang, Yanru Xue, Xiaogang Wu, Weiyi Chen, Liming He

**Affiliations:** ^1^ College of Artificial Intelligence, Taiyuan University of Technology, Taiyuan, China; ^2^ Beijing Aeronautical Technology Research Center, Beijing, China; ^3^ Shanxi Key Laboratory of Bone and Soft Tissue Injury Repair, Department of Orthopaedics, The Second Hospital of Shanxi Medical University, Taiyuan, China; ^4^ Shanxi Bethune Hospital, Shanxi Academy of Medical Sciences, Tongji Shanxi Hospital, Third Hospital of Shanxi Medical University, Taiyuan, China

**Keywords:** femoral neck fracture, internal fixation design, finite element modeling, biomechanical analysis, porous structure

## Abstract

Prolonged internal fixation in the femur can cause issues like osteosclerosis and stress masking, which hinder fracture recovery. AZ31b magnesium alloy has already been widely investigated and shown to be biocompatible and biodegradable. This study used finite element analysis to investigate stress changes during healing, aiming to find the best time for removing internal fixation and focuses on optimizing the structural design of internal fixation, using AZ31b magnesium alloy, to address issues like osteosclerosis and stress shielding in femoral neck fracture healing. This study focuses on optimizing structural design for the treatment of Pauwels III femoral neck fractures using cannulated screws. Pauwels III fractures are characterized by high shear stress and unstable fracture angles, making them prone to fixation failure. By optimizing the fixation method, the risk of complications such as osteosclerosis and stress shielding can be mitigated, ultimately improving clinical outcomes. The findings show that when cancellous bone heals but cortical bone does not, stress on the fracture surface decreases. Early removal of titanium internal fixation, followed by biodegradable porous internal fixation, allows for natural degradation during healing. We simulated stress evolution across healing stages via finite element modeling to determine optimal fixation replacement timing. Four AZ31b magnesium alloy porous structures (cubic, honeycomb, diagonally orientated, modified truncated pyramid) were designed; their equivalent elastic modulus and strength were evaluated through simulated compression tests, while permeability was analyzed using computational fluid dynamics (CFD). We found that the cubic and honeycomb structures were found to have higher permeability (6.23 × 10^-7^ m^2^, 1.636 × 10^-7^ m^2^) and have high elastic modulus (8.422Gpa, 14.694Gpa) which can match the elastic modulus of human bone. Optimal structures were then applied to an inverted triangular screw group model for biomechanical validation post-femoral neck fracture fixation. Finite element analysis of a Pauwels III femoral neck fracture model indicated that the honeycomb porous internal fixation had superior mechanical properties. In conclusion, this study proposed a solution to osteosclerosis and stress masking after femoral neck fracture surgery.

## 1 Introduction

The skeleton is an organ undergoing constant biological remodeling, experiencing dynamic changes within the body. During this process, osteoclasts absorb aging bone tissue, while osteoblasts generate new bone tissue, maintaining the internal environment’s balance (Wang et al., 2016; Huawei et al., 2019). The process of fracture healing is highly complex. In recent years, numerous new technologies, such as immunological techniques, histology, genetic engineering, and electron microscopy, have been applied to investigate the mechanisms of fracture healing. These studies have provided a deep understanding of many aspects of fracture healing, although some mechanisms remain incompletely understood. According to Wolff’s law, prolonged exposure to external stress can cause changes in the internal and external structure of the bone to adapt to new environmental conditions. Mechanical loading plays a dual regulatory role in the process of trabecular bone formation. During the initial stage of femoral neck fracture healing, moderate mechanical loading plays crucial in remodeling trabecular bone and gradually maintaining stability to ensure certain performance (Lambers et al., 2013). However, if the trabecular bone is unable to withstand the mechanical loading exerted on it, severe damage and deformation may occur (Lin et al., 2020), eventually leading to necrosis and collapse, resulting in femoral head osteonecrosis. Sclerosis around the screw channel occurs due to stress shielding, where the higher stiffness of metallic implants reduces the mechanical load on the surrounding bone, impairing its ability to remodel and regenerate. Studies have shown that even after the healing of femoral neck fractures if the area of sclerosis around the screw channel persists, there is still a probability of collapse of the femoral head even after the removal of internal fixation implants. Even after many years of removing the internal fixation materials, there will be no effective bone tissue filling in the screw channel. Therefore, preventing the formation of the sclerotic zone in the bone is an important area for further research (Liu et al., 2023).

During the treatment of fractures, the use of plates and screws may lead to bone sclerosis near the screw channel, a condition known as screw channel bone sclerosis. This sclerosis can delay the healing process of the fracture, posing challenges to the treatment. Therefore, current researchers are actively developing and selecting various biomaterials in the medical field to reduce bone sclerosis around the screw channel while promoting bone healing. The selection of these materials is primarily based on their superior biocompatibility and ability to promote bone tissue regeneration. For instance, bioactive glass has garnered widespread attention due to its excellent bioactivity and ability to bond with bone (Zheng et al., 2021), while bioceramics and magnesium alloy materials have also gained significant attention for their good biocompatibility (Miri et al., 2024; Zhang et al., 2023). These materials not only provide structural support to the fracture site but also interact with the human skeletal system, promoting the recovery of bone defects and bone remodeling. With the continuous progress in the field of biomaterials, these innovative materials hold tremendous potential for use in orthopedic surgeries and are expected to significantly enhance the effectiveness of fracture treatment. Some studies have explored the use of bioactive substances to promote fracture healing and reduce screw channel bone sclerosis. These bioactive substances mainly include growth factors and bone morphogenetic proteins, which play a crucial role in the process of bone repair and reconstruction (Wang et al., 2023). Typically, these bioactive substances are administered directly to the site of the fracture or combined with other carrier materials such as collagen and hydroxyapatite to enhance their stability and bioactivity at the target site (Kon et al., 2010). However, the clinical application of these methods still faces several challenges, such as dosage control, long-term stability, and issues of biocompatibility and safety.

Pauwels type III femoral neck fractures are highly unstable due to their steep fracture angle, leading to high shear stress and a higher risk of fixation failure. This study focuses on optimizing the structural design of internal fixation specifically for Pauwels type III fractures. While AZ31b magnesium alloy has been well-documented for its biocompatibility and controlled degradation, the research gap lies in the lack of optimized fixation strategies for these fractures that both minimize stress shielding and promote bone healing through gradual degradation (Zhang et al., 2025). This study aims to address this gap by focusing on both structural design optimization and material selection. This study examines the stress conditions on the fracture surface during the process of fracture healing, aiming to determine the optimal timing for removing internal fixation to prevent the formation of bone sclerosis. Suitable material (AZ31b magnesium alloy) was selected to meet the strength requirements for treating femoral neck fractures while being biodegradable, allowing for the degradation of the internal fixation material as the screw channel is filled. Four different porous structures were chosen, and their mechanical properties were compared to determine the most suitable porous structure for application in an inverted triangle screw group. Subsequently, the feasibility of the proposed implantation scheme was evaluated following its implantation into the fractured femur.

## 2 Materials and methods

### 2.1 Studies of fracture surface healing

Femoral neck fractures are commonly treated by implanting internal fixation. However, due to the prolonged implantation period, there is a risk of sclerosis in the screw channel when removing the internal fixation after the fracture surface has completely healed. This hinders inward bone growth and increases the instability and probability of collapse and necrosis in the femoral head. Therefore, this study aims to identify an appropriate timing during the fracture healing process. At this point, the femur has regained sufficient load-bearing capacity, reducing its dependence on internal fixation. Although the fracture surface has not yet fully healed, solid internal fixation can be removed early and replaced with a novel porous internal fixation made of biodegradable materials. This new internal fixation provides a certain degree of support to the femur and allows for the transportation of nutrients between the pores. As the fracture surface continues to heal, the bone gradually fills the screw channel through the pores, while the new internal fixation degrades. This effectively prevents the occurrence of channel sclerosis. This approach of initially using solid internal fixation until the femur has acquired some load-bearing capacity and then transitioning to biodegradable internal fixation also addresses the issue of early failure that can arise from using biodegradable internal fixation from the beginning, where degradation occurs too rapidly.

**FIGURE 1:**
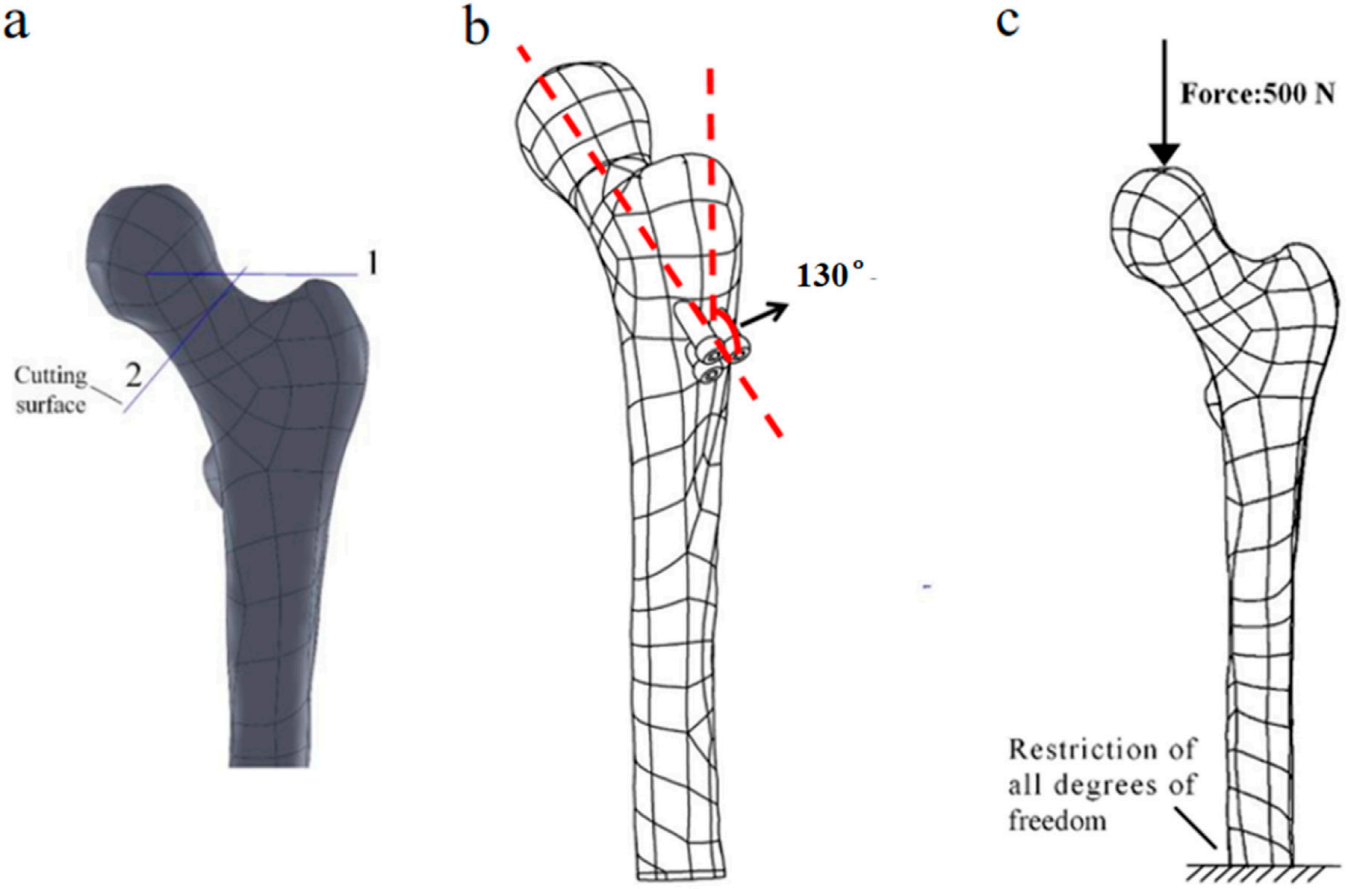
**(a)** Femoral fracture model; **(b)** Inverted triangular screw group implantation in femur; **(c)** Boundary conditions.

**FIGURE 2:**
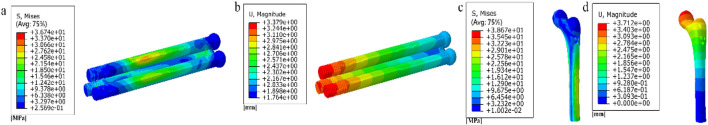
**(a)** Stress distribution of the inverted triangle screw group; **(b)** Displacement distribution of the inverted triangle screw group; **(c)** Stress distribution of the femur; **(d)** Displacement distribution of the femur.

First, Create a femoral neck fracture model: Select one healthy male volunteers aged 30-40 to sign an informed consent form. Perform a 64 slice multi-slice spiral CT (GE, USA) scan from the upper femur to the middle tibia to obtain a two-dimensional cross-sectional image with a layer thickness of 1.0 mm. Store the CT image as a digital imaging and medical communication (DICOM) format file and import it into Mimics 19.0 (Materialise, Belgium). Separate bones from other tissues through threshold partitioning, create a separate left femur using a separation mask, and reconstruct the 3D entity of the left femur through mask editing, cavity filling, and other operations. Save the file as an STL (stereolithography) format file and import it into Geomagic Wrap 2017 (Oqton, USA) for surface smoothing treatment, construction of surface patches, construction of grids, fitting of surfaces, and construction of cortical and cancellous bones. Export the model as an STP (Product Data Model Standard Interchange) format file and import it into Solidworks 2018 (Dassault Systemes, France). A hollow triangular screw assembly and a femur model with femoral neck fracture were established in Solidworks 2018. Construct a three-dimensional model of Pauwels III femoral neck fracture by creating a plane one parallel to the horizontal plane of the human body and a plane two intersecting the plane. The angle between the two planes is 50°, and plane two is the cutting surface of the fracture, as shown in Figure 1a. The screws were inserted at a 130° angle relative to the femoral axis, as commonly used in clinical practice for Pauwels III fractures, as shown in Figure 1b. Construct a three-dimensional model of Pauwels III femoral neck fracture by creating a plane one parallel to the horizontal plane of the human body and a plane two intersecting the plane. screw diameter was 6mm, and the two were assembled. The intact femoral model was reconstructed in SolidWorks, meshed in Hypermesh, and validated against displacement and stress distributions from published studies. The model was then meshed in Hypermesh 14.0 using tetrahedral elements. The number of elements for the hollow triangular screw assembly was 28,710, with 9,224 nodes. The cortical bone had 27,727 elements and 9,360 nodes, while the cancellous bone had 49,727 elements and 11,358 nodes. The mesh model was imported into Abaqus 6.14 for finite element analysis with boundary conditions shown in Figure 1c. The material properties of the model are listed in Table 1. The results are presented in Figure 2, and they were compared with previous research results (Yang, 2022). The peak displacement of the femur in this study is 3.712 mm, which is similar to the femur displacement of 3.129 mm in the reference literature and the distribution of the femur model were similar, confirming the validity of the model.

**TABLE 1:** Material properties of finite element model.

Composition	Young’s modulus (MPa)	Poisson’s ratio
Femoral neck cancellous bone	620	0.29
Cancellous bone of the femoral head	840	0.29
Intertrochanteric cancellous bone of the femur	300	0.29
Femoral cortical bone	16,800	0.3
Titanium alloy	110,000	0.33

**FIGURE 3:**
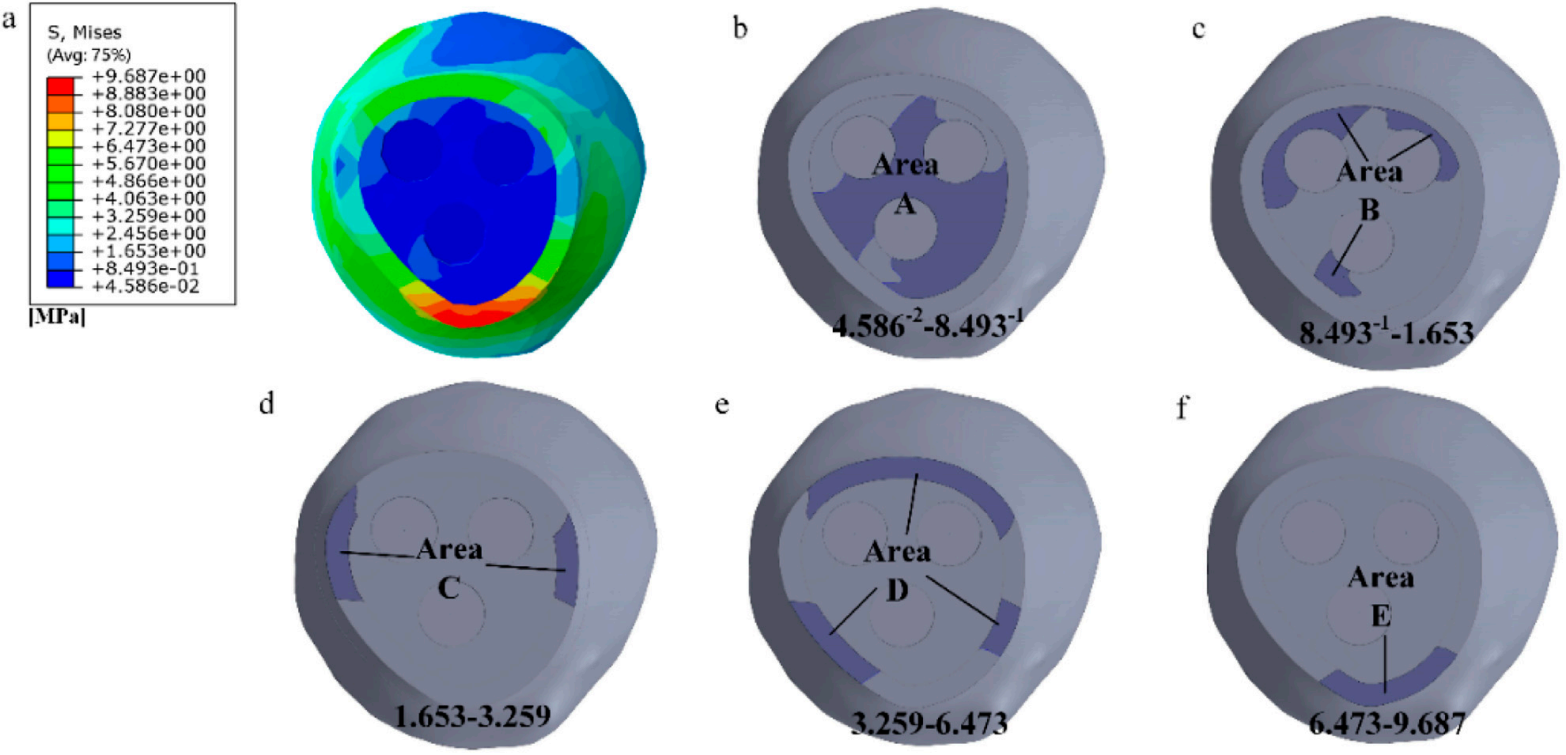
Delineating healing areas: **(a)** Fracture surface stress diagram; **(b–f)** Different stress areas from low to high.

As shown in Figure 3a, by observing the stress distribution on the fracture surface, distinct stress zones can be identified. Previous studies have found that fracture healing is influenced by various factors, including (especially) mechanical stability, soft tissue and vascular damage, as well as patient complications (Armiento et al., 2020). Based on this research, the fracture surface can be divided into five regions (A-E) based on different stress levels, as illustrated in Figures 3b–f. Regions with lower stress exhibit better mechanical stability and faster recovery, while regions with higher stress demonstrate poorer mechanical stability and slower recovery. Region A experiences the least amount of stress, thus it can be prioritized for healing. In this case, the proximal and distal ends of the femoral fracture surface in region A are combined in Solidworks, while the other regions remain uncombined. The partially healed femur model is then analyzed in Abaqus. In the staged healing simulation, fully fixed constraints are applied to the healed area to simulate bone fusion; The unhealed area retains frictional contact (friction coefficient = 0.3), allowing relative displacement to reflect incomplete biological healing. This method reproduces the healing process of mechanically stable areas prioritizing ossification in clinical practice. After obtaining the results, the region with the second lowest stress on the fracture surface, region B, is selected for healing, following the same procedure as in region A. This process continues until region E is healed, indicating complete healing of the fracture surface. The hypothesis of implanting stents at this specific healing stage was chosen in this study, based on the unique healing biology of cancellous bone. Unlike cortical bone that relies on endochondral ossification, cancellous bone fractures mainly heal through rapid “trabecular bone formation”, which is a direct intramembranous ossification method that can initiate within a few days after injury and form a woven bone network with initial load-bearing capacity within a few weeks (Sandberg and Aspenberg, 2016). Pauwels III femoral neck fracture, as a typical cancellous bone fracture, has this characteristic in its early healing. Therefore, what this study seeks through finite element simulation is precisely the “time window” during which this initial callus has critical mechanical support capabilities and allows for the intervention of degradable scaffolds. The stress distribution on the fracture surface at different stages of healing is observed. In clinical practice, the continuity of the bone can be visualized through X-ray, while CT scans can be used to observe changes in bone density. Additionally, various measurement techniques, including ultrasound, direct static measurement, and vibration measurement, can be employed to assess fracture healing status (Najafzadeh Biomechanical assessment and quantification, 2022).

### 2.2 Creation of porous units

Metal, as an implant material, typically exhibits a significantly higher elastic modulus than the surrounding bone tissue. This difference results in relatively high stress in the area where the metal is in contact with the bone, and stress shielding occurs once the metal is implanted into the human body. This phenomenon may further induce slow bone recovery and negative reactions such as implant loosening. To address this issue, the application of a porous structure in internal fixation can effectively reduce the overall stiffness of the implant, allowing it to better adapt to the surrounding bone tissue environment. Additionally, while metal implants themselves are not easily degradable, their porous structure creates conditions for cell proliferation, facilitating the growth of bone cells. This allows bone tissue to penetrate and grow within these interconnected pores, forming a secure and close connection with the implant, thereby enhancing the overall stability of the implant. Designing implants with a porous nature can address numerous challenges encountered after surgery, such as aseptic loosening and infection issues. Therefore, the porous structure becomes an indispensable element in the design of orthopedic implants.

**FIGURE 4:**
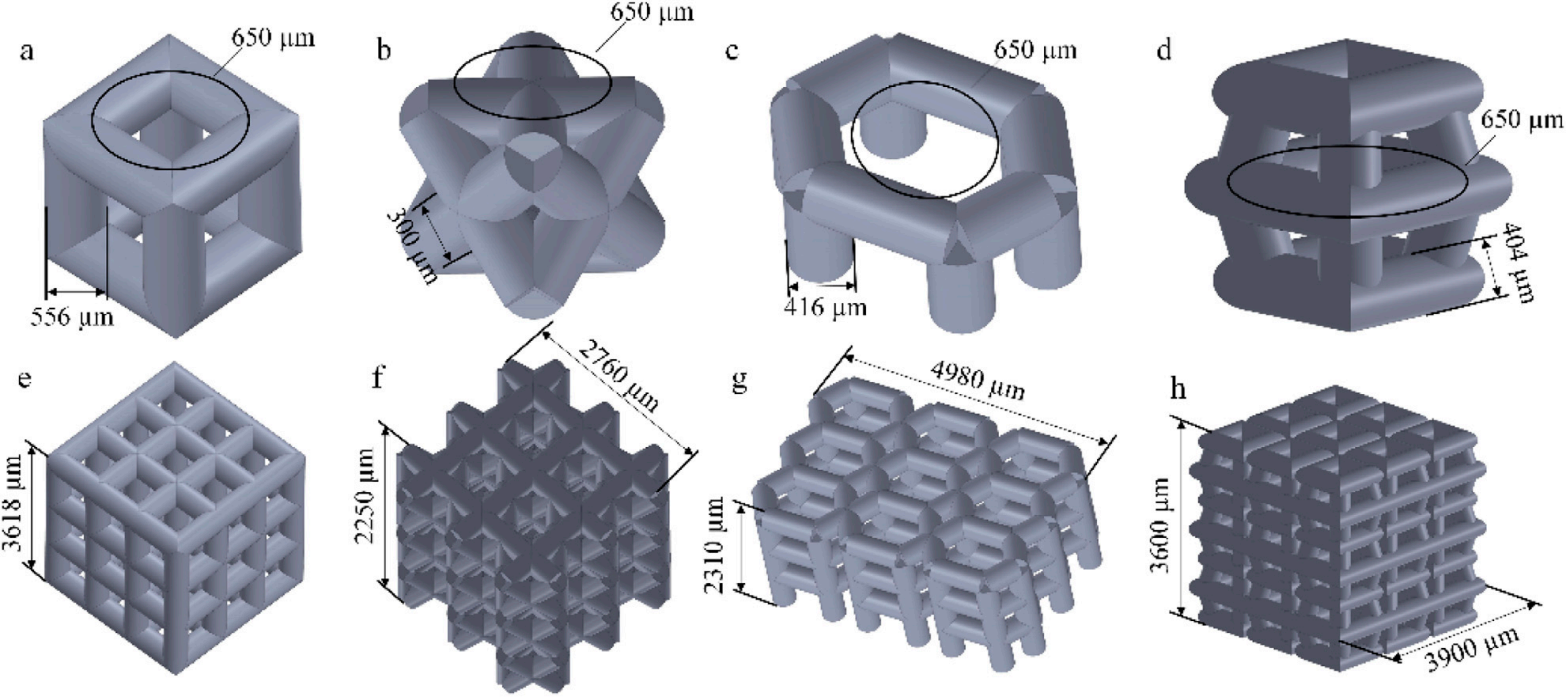
Four porous structures and their 3 × 3 array sets: **(a)** cubic unit; **(b)** diagonally orientated unit; **(c)** honeycomb unit; **(d)** modified truncated pyramid unit; **(e–h)** Array of units.

We selected four porous structures: cubic unit, diagonally orientated unit, honeycomb unit, and modified truncated pyramid unit, as shown in Figure 4. These structures have been extensively studied and applied in other fields, such as hip joint prostheses and bone grafts, demonstrating their reliable performance and suitability for use in bone implants (Murr, 2017; Wieding et al., 2014).

The parameters for establishing porous structures primarily consider pore size, strut diameter, and porosity. Current research indicates that structures with high porosity play a promoting role in the internal growth of bone tissue. There are still different viewpoints in the academic community regarding the selection of pore size for better promotion of bone tissue growth. However, it is generally believed that micropores within the range of 100 μm–900 μm are conducive to cell growth. Structures with smaller pore sizes have larger surface areas, providing more favorable space for the inward growth of bone tissue. Smaller pore sizes may also benefit the enhancement of the mechanical performance of porous scaffolds (Zhao et al., 2018). Guo et al. fabricated porous Ti6Al4V implants using SLM and compared the performance of implants with pore sizes of 300 μm, 500 μm, and 700 μm. In the groups of implants with pore sizes of 500 μm and 700 μm, superior collagen tissue development and cell alignment were observed, and better vascular formation was observed in both groups (Guo et al., 2022). Naoya’s team designed three titanium porous scaffolds with pore sizes of 309 μm, 632 μm, and 956 μm and implanted them in rabbits for 12 weeks. The results showed that the scaffold with a pore size of 632 μm outperformed the other two, exhibiting higher compressive strength and demonstrating stronger fixation after implantation (Taniguchi et al., 2016). Based on previous experience, the pore size was determined as 650 μm in this experiment.

Porosity refers to the ratio of the volume of the porous portion of the structure to the total volume of the structure, and its calculation is according to Formula 1.

$$P=\frac{V_{0}-V}{V_{0}}\times 100\%$$ (1)

In the above formula, P is the porosity of the porous structure, V0 refers to the total volume of the structure, and V refers to the volume of the porous scaffold.

Lv et al. utilized the finite element method to investigate the relationship between Young’s modulus of nanosilver and porosity. The results indicated that Young’s modulus decreased with increasing porosity. When the porosity ranged from 40% to 60%, the structural elastic modulus was found to be 5.2–19.94 GPa, which falls within the range of the elastic modulus of human bones (Lv et al., 2023). Simoneau et al. suggested that a porosity range of 30%–50% in porous structures maximizes the surface area-to-volume ratio, thereby promoting bone regeneration (Simoneau et al., 2017). Based on these findings, this study selected a porosity rate of 45% as an indicator for porous structures.

The manufacturing range of pore size for porous structure pillars using SLM technology is limited to 200–1,500 μm (Wieding et al., 2014). Based on the determined pore size and porosity mentioned above, the pillar diameter can be calculated using the porosity formula, with the requirement that the pillar diameter falls within the range of 200–1,500 μm. The results are presented in Table 2.

**TABLE 2:** Parameters of four porous structures.

Parameters\structures	Cubic	Diagonally orientated	Honeycomb	Modified truncated pyramid
Pore size (μm)	650	650	650	650
Porosity	44.9%	45%	45%	44.9%
Pillar diameter (μm)	556	300	416	404
Array	3 × 3 × 3			

### 2.3 Selection of degradable materials

Currently, the majority of bone implant materials are made of metals, which possess a high elastic modulus causing stress shielding in the bone. Moreover, their biocompatibility is relatively poor, particularly during long-term usage. In certain cases, certain metal implants may even release harmful ions, which are detrimental to bone growth.

Magnesium ions are abundant cations in the human body and play multiple essential roles. They not only serve as cofactors for over 300 enzymatic reactions, facilitating various metabolic processes, but also contribute significantly to the maintenance of cognitive function in the elderly. Studies have shown a close correlation between magnesium ions and brain function (Xu et al., 2021). Magnesium ions demonstrate excellent degradability in human blood, and when the chloride ion concentration is high, complete degradation of magnesium ions can occur. The widespread clinical application of magnesium further confirms its reliability as a degradable metal material. Research suggests that magnesium ions generated from the degradation of magnesium compounds may possess certain anti-thrombotic functions (Mahanty and Shikha, 2023), potentially due to their ability to reduce the deposition of inflammatory cells and platelets. Magnesium alloy exhibits excellent biocompatibility. For example, Yuan, G.Y. et al. implanted an internal fixation system made of JDBM magnesium alloy into rabbit bone and confirmed its *in vivo* performance. After a certain period of time, no significant inflammation or foreign body reactions were observed. The experimental rabbits exhibited normal indicators and were unaffected by the magnesium alloy internal fixation system (Kong, 2015). Additionally, compared to degradable polymer materials, magnesium alloys as degradable scaffold materials offer superior mechanical properties.

AZ31b magnesium alloy has attracted significant attention as a structural material due to its low density, lightweight per unit volume, and environmentally friendly characteristics. Currently, magnesium alloys are gradually replacing aluminum alloys in various fields (Sun et al., 2023). The elastic modulus of AZ31b is 44.8 GPa, with a Poisson’s ratio of 0.35, making it less stress shielding compared to Titanium alloy (Ti6Al4v) alloys (110 GPa).

### 2.4 Simulated compression experiment

When designing porous structures, two main aspects need to be considered. Firstly, the porous structure must ensure sufficient strength and stiffness to avoid damage under load after surgical implantation. Simultaneously, this structure should possess an elastic modulus similar to that of human bones to prevent stress shielding. Secondly, the porous structure should exhibit good permeability with appropriately sized pores to facilitate the diffusion of nutrients and aid in the removal of metabolic waste. Based on these design requirements, compression tests and permeability analysis experiments were conducted on four types of porous structures in this section and the following section.

**FIGURE 5:**
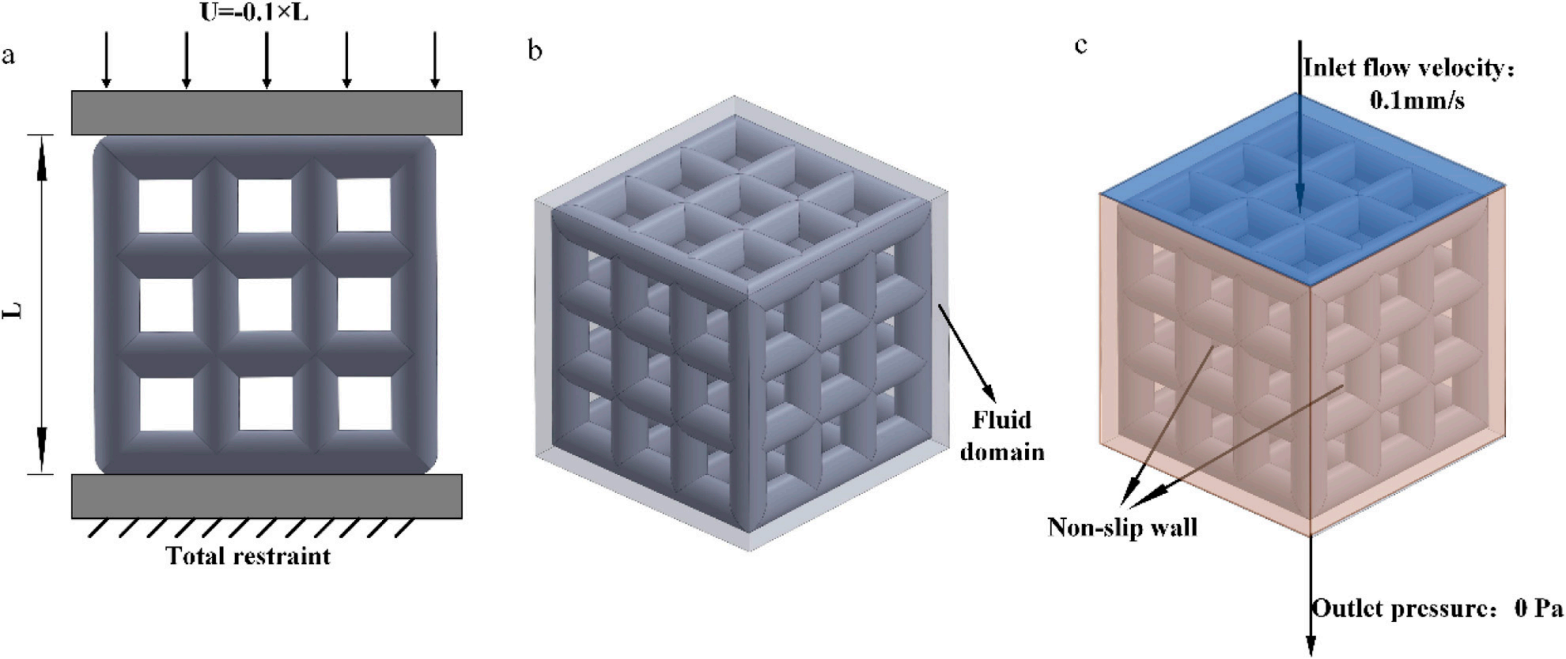
**(a)** Simulated compression; **(b)** Fluid domain; **(c)** Fluid simulation boundary conditions.

The simulation experiment, as shown in Figure 5a, involved the compression of the porous structure using two rigid bodies. A reference point was established in Abaqus, by constraining the degrees of freedom of the entire model through the degrees of freedom of the reference point, the compressive behavior of the model can be represented by extracting the force-displacement data of the reference point from the computational results. The compression displacement is approximately 10% of the structural length in the compression direction (Wieding et al., 2014). The stress-strain values are calculated according to Formula 2 and Formula 3.

$$\varepsilon =\frac{U}{L}$$ (2)

$$\sigma =\frac{F}{S}$$ (3)

In the above formula, ε represents the strain of the porous structure, U denotes the displacement in the compression direction of the structure, L represents the length in the compression direction of the structure, δ signifies the stress of the porous structure, F represents the reaction force on the lower surface of the structure, and S denotes the bottom surface area of the structure.

### 2.5 Permeability analysis

We transformed the solid structure into a porous structure with the aim of not only reducing its elastic modulus but also ensuring sufficient permeability. This permeability plays a crucial role in cell proliferation, differentiation, and bone regeneration processes. Previous research has shown that porous bone scaffolds with higher permeability exhibit excellent cellular activity, as their pores facilitate cell adhesion and migration. Consequently, they possess significant potential for development and application (Zhao et al., 2022). Therefore, to ensure comprehensive performance after implantation in the human body, it is essential to select units with higher permeability as the foundational structure.

We employed the computational fluid dynamics (CFD) method to simulate the process of four different porous structures in a fluid domain. According to fluid mechanics theory, this process can be described using the Navier-Stokes equations (Formula 4):

$$\rho \frac{\partial u}{\partial t}-\mu \nabla^{2}u+\rho \left(u*\nabla \right)u+\nabla P=F$$ (4)

The following variables are used: ρ represents the fluid density (kg/m3), u denotes the fluid velocity (m/s), t signifies time, μ represents the fluid viscosity coefficient (Pa·s), P represents the pressure (Pa), and F represents the external force (N).

The fluid domain in this study was simulated using Dulbecco’s Modified Eagle Medium (DMEM), with a dynamic viscosity coefficient of 0.00145 Pa s and a density of 1,000 kg/m^3^ (Singh et al., 2018). Using 3D software to establish a pure flow field model. In Abaqus, the model was set with an inlet velocity of 0.1 mm/s and an outlet pressure of 0 Pa. The inlet flow rate of 0.1 mm/s is designed to simulate the physiological rate of nutrient transport in bone tissue, consistent with the low-speed fluid dynamics simulation parameters used for stent permeability testing. This value conforms to the *in vitro* study range of interstitial fluid flow velocity in bone trabeculae, ensuring biological relevance (Olivares et al., 2009). The surfaces surrounding the pure flow field were defined as non-slip walls, and the flow direction was set as top-down, as shown in Figures 5b,c. The flow behavior was explained by Darcy’s law, according to Formula 5.

$$K=\frac{V\mu L}{\Delta P}$$ (5)

The following parameters were used: K - permeability; V - fluid inlet velocity (m/s); μ - dynamic viscosity of DMEM (Pa·s); L - height of the structure in the direction of fluid flow (m); ΔP - pressure difference between the inlet and outlet. The pressure drop values were obtained from finite element calculations, while the remaining parameters were known. This allowed for the calculation of the permeability of the porous structure.

### 2.6 Study on the treatment of femoral neck fracture with degradable porous internal fixation

We conducted simulated compression experiments and permeability analysis on four different porous structures. Based on the results, we ultimately selected the cubic and honeycomb structures for application in internal fixation devices. These structures have equivalent elastic moduli within the range of human bone elastic moduli, which can reduce the occurrence of stress shielding. Additionally, they exhibit higher permeability compared to the other two structures. When applied in internal fixation, nutrients can flow more rapidly between the pores. Furthermore, their lower peak pressure drop and flow velocity are conducive to cell adhesion and growth on the surface of the structures.

**FIGURE 6:**
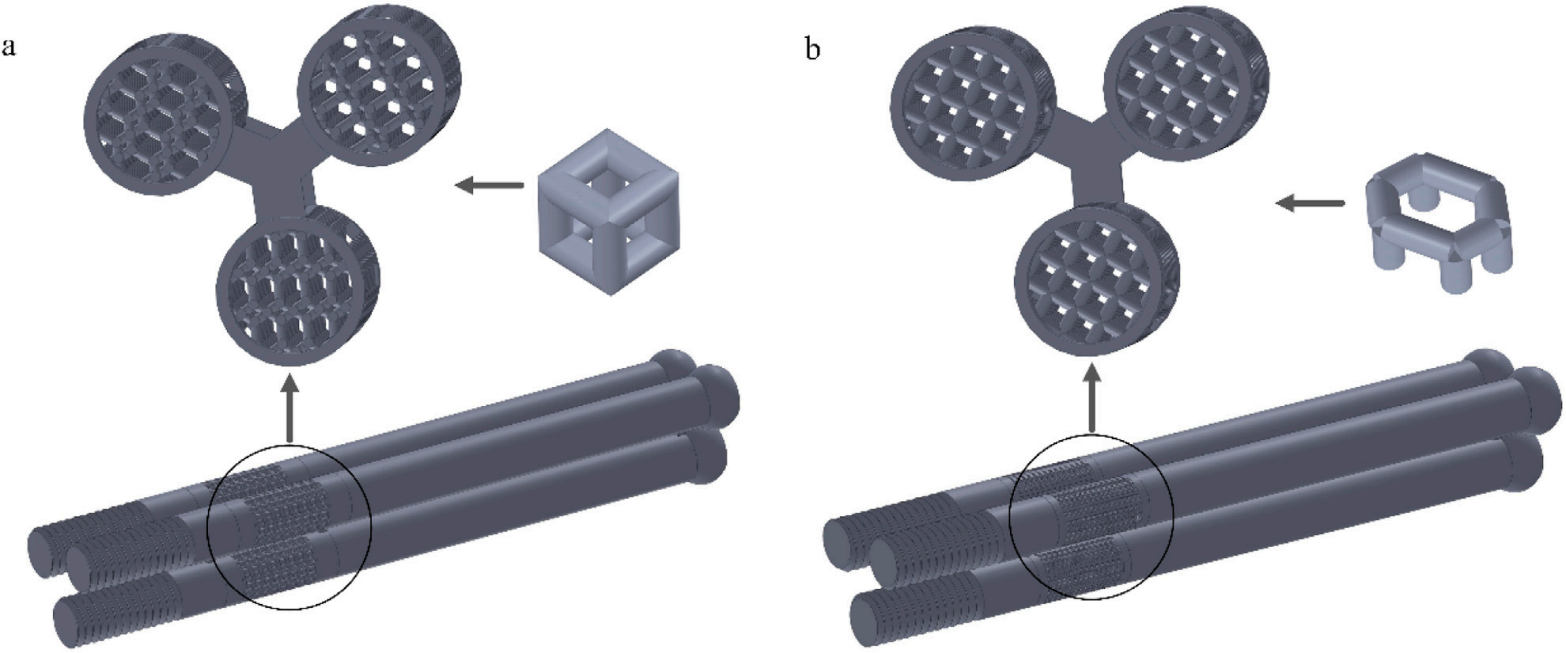
Internal fixation of 10 mm segment porous inverted triangle screw: **(a)** Cubic segmental porous internal fixation; **(b)** Honeycomb segmental porous internal fixator.

**FIGURE 7:**
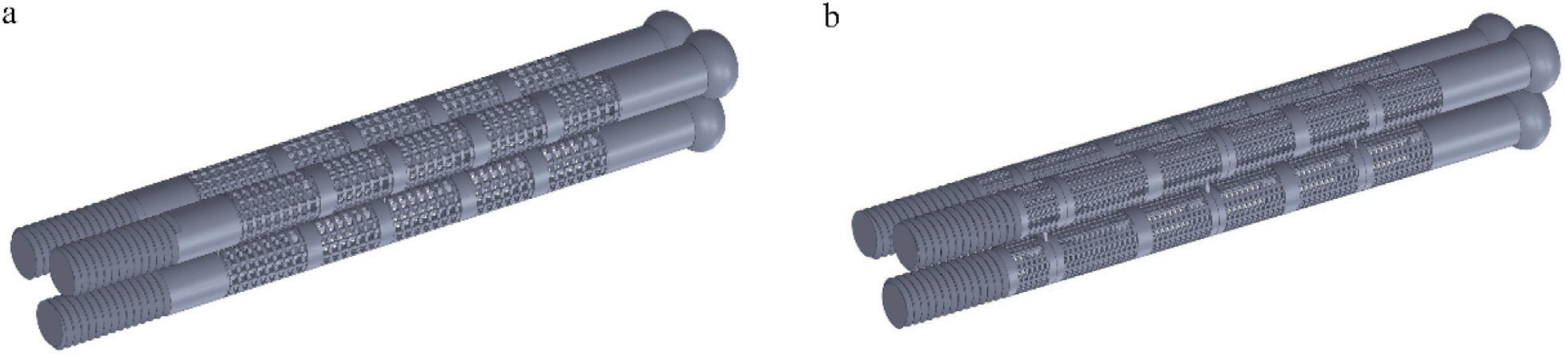
Internal fixation with fully porous inverted triangle screw: **(a)** Cubic fully porous internal fixation; **(b)** Honeycomb fully porous internal fixation.

Internal fixation with an inverted triangle screw group is one of the primary means used in the clinical treatment of Pauwels type III femoral neck fractures. During clinical procedures, screw implantation can be performed under intraoperative fluoroscopic guidance. A minimally invasive incision is made at the greater trochanter to insert a guidewire. After closed reduction is completed, three connected biodegradable porous magnesium alloy hollow screws are implanted along the guidewire at a 130-degree angle in an inverted triangular configuration to fix the femoral neck fracture. This inverted triangular configuration, along with the structural design of the screws, enables compression between fracture fragments. It provides stable support for Pauwels type III fractures and promotes bone ingrowth through its porous structure, thereby avoiding the need for a secondary removal surgery (Yalin et al., 2023). Due to its long-standing clinical application and high reliability, we chose to apply a porous structure to this type of internal fixation. Based on previous studies on fracture healing stages, we healed the cancellous bone, that is, the proximal fracture piece was connected with the distal femur, and the cortical bone was still in a state of fracture. Considering the significant number of grids required for dividing the porous structure and the time-consuming nature of running finite element software, we conducted a preliminary test. We designed a 10 mm-long porous structure for the portion of the internal fixation near the fracture line, as this area experiences higher shear forces. If the results were within the normal range and showed no significant deformation, we would proceed to apply the porous structure to the entire internal fixation, as shown in Figure 6. By comparison, the finite element results are close to the model validity verification results, so the porous structure is applied to the whole internal fixation, The screw’s threaded section, responsible for providing fixation, remained non-porous, while the screw’s end predominantly resided outside the femur, remained non-porous, as shown in Figure 7. Boundary conditions remained consistent with the preceding content. Tetrahedral grids were employed for partially porous models. The cubic internal fixation units consisted of 2,454,223 elements with 594,923 nodes, while the honeycomb internal fixation units comprised 2,725,579 elements with 684,139 nodes. The cortical bone consisted of 183,921 elements with 49,191 nodes, while the cancellous bone consisted of 432,747 elements with 101,920 nodes. In the complete porous model, the cubic internal fixation units reached 3,379,050 elements with 878,215 nodes, while the honeycomb internal fixation units totaled 8,308,357 elements with 2,016,223 nodes. The cortical bone consisted of 183,921 elements with 49,191 nodes, while the cancellous bone comprised 432,747 elements with 101,920 nodes.

## 3 Results

### 3.1 Findings on the healing of fracture surfaces

**FIGURE 8:**
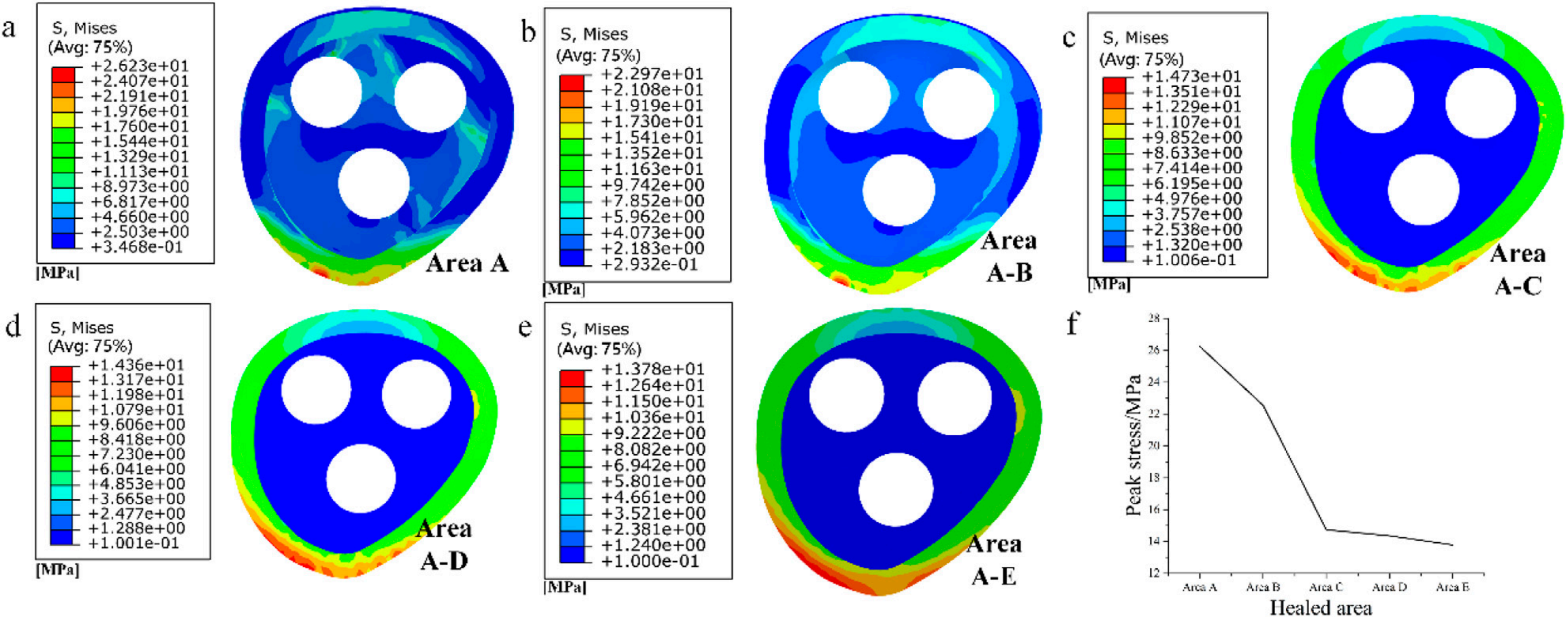
**(a–e)** Stress distribution of fracture surface from area A to area E during progressive healing; **(f)** Peak stress in different healed areas

As shown in Figure 8, the stress distribution in different stages of fracture healing can be observed from the stress contour diagram. The peak stress on the fracture surface occurs in the lower part of the femoral neck near the talus, both during cancellous and cortical bone healing. During the healing process in cancellous bone, regions C and D on the cortical bone experience relatively lower stress, while during the healing process in cortical bone, regions A and B on the cancellous bone experience relatively lower stress. The load is primarily borne by the cortical bone. Comparing the peak stress values on the fracture surface in this experiment with the data from stable fractures under the same conditions with various implanted internal fixations, which ranged from 5 to 17 MPa (Zhang et al., 2017), it is found that the peak stress in region C during the healing process is within this “safe range”. Moreover, compared to the previous healing stage the stress on the fracture surface is significantly reduced. And it is not difficult to see that after the healing of region C, the peak stress values of regions D and E hardly decreased during the healing process. Therefore, during the healing process in region C, the fracture surface already possesses sufficient load-bearing capacity. It can serve as a crucial symbol for the clinical removal of internal fixations and the implantation of degradable internal fixations simultaneously, providing support for the recovery of the remaining non-healed portion and promoting its restoration.

### 3.2 Simulated compression test results

**FIGURE 9:**
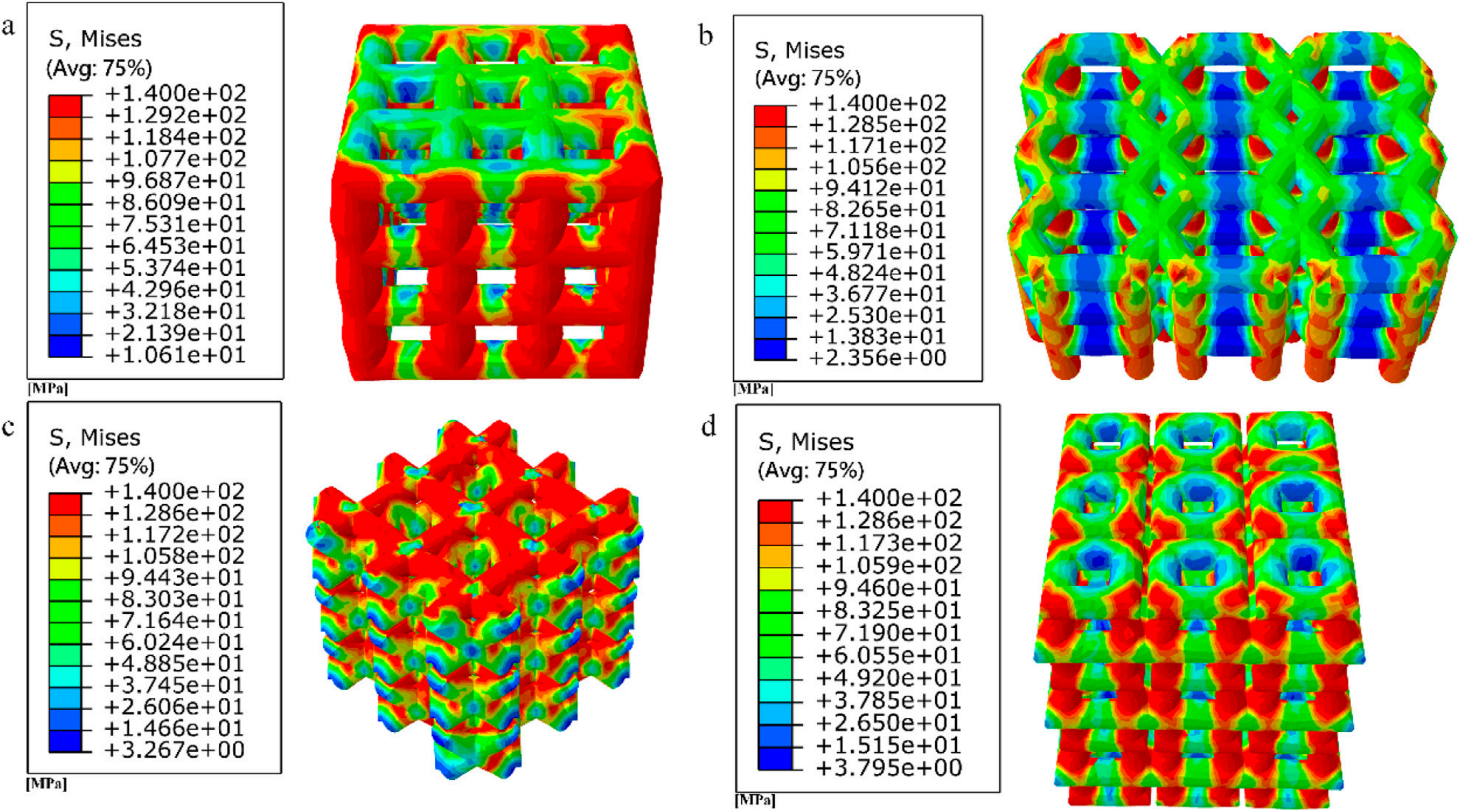
Simulated compression experimental results for four porous structures: **(a)** cubic structure; **(b)** honeycomb structure; **(c)** diagonal orientated structure; **(d)** modified truncated pyramid structure.

**FIGURE 10:**
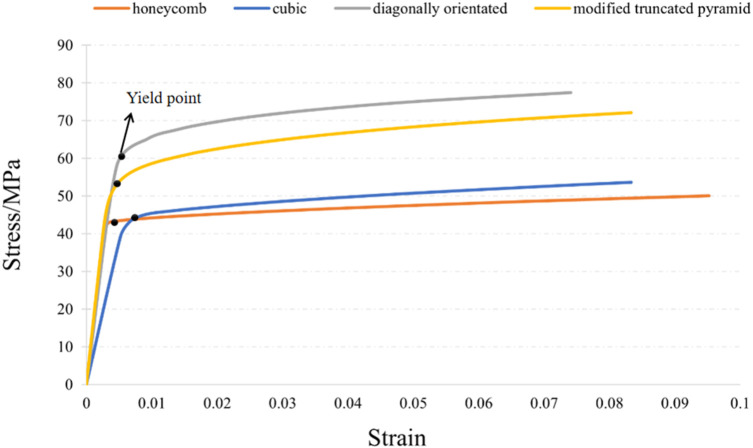
Stress-strain curve.

The compression results of the four porous structures are shown in Figures 9, 10, and the equivalent modulus of elasticity and equivalent strength of the four porous structures are summarized in Table 3.

**TABLE 3:** Summary of equivalent modulus of elasticity and equivalent strength of four porous structures.

Parameters\structures	Cubic	Diagonally orientated	Honeycomb	Modified truncated pyramid
Compression direction length (mm)	3.6	2.3	2.3	3.6
Compressive displacement (mm)	0.3	0.2	0.2	0.3
Equivalent young’s modulus (GPa)	8.422	12.513	14.694	15.809
Equivalent strength (MPa)	53.64	77.44	50.02	72.12

The equivalent modulus of elasticity was the smallest for the cubic structure at 8.422 GPa and the largest for the modified truncated pyramid structure at 15.809 GPa; the equivalent strength was the smallest for the honeycomb structure at 50.02 MPa and the largest for the diagonally orientated structure at 77.44 MPa.

### 3.3 Results of permeability experiments

**FIGURE 11:**
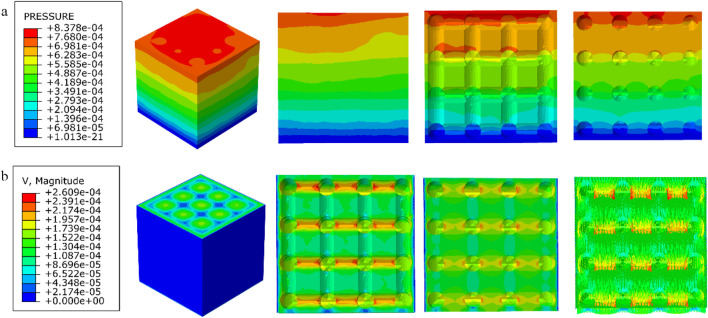
**(a)** Wall shear stress distribution of cubic structure; **(b)** pore flow rate distribution of cubic structure.

**FIGURE 12:**
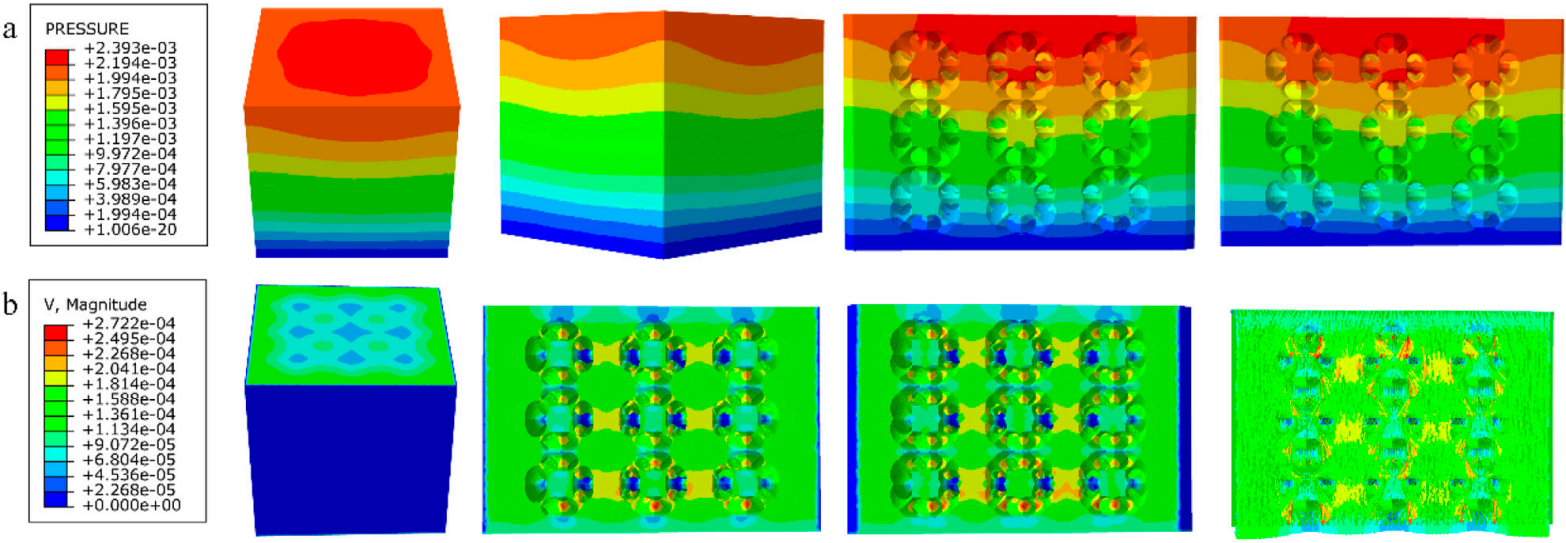
**(a)** Wall shear stress distribution of diagonally orientated structure; **(b)** pore flow rate distribution of diagonally orientated structure.

**FIGURE 13:**
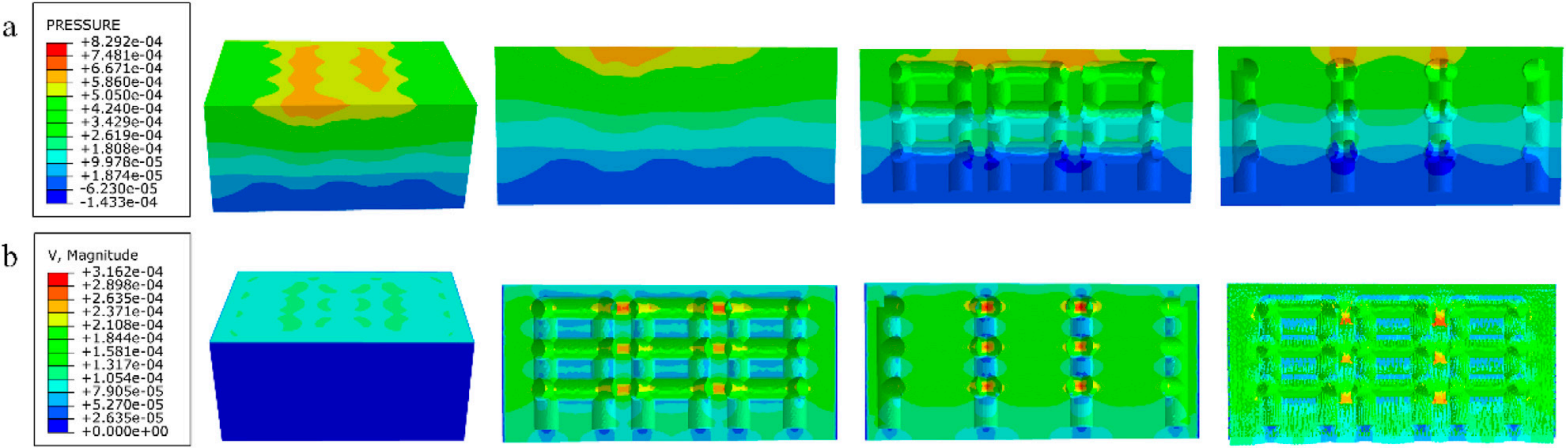
**(a)** Wall shear stress for honeycomb structure; **(b)** pore flow rate for honeycomb structure.

**FIGURE 14:**
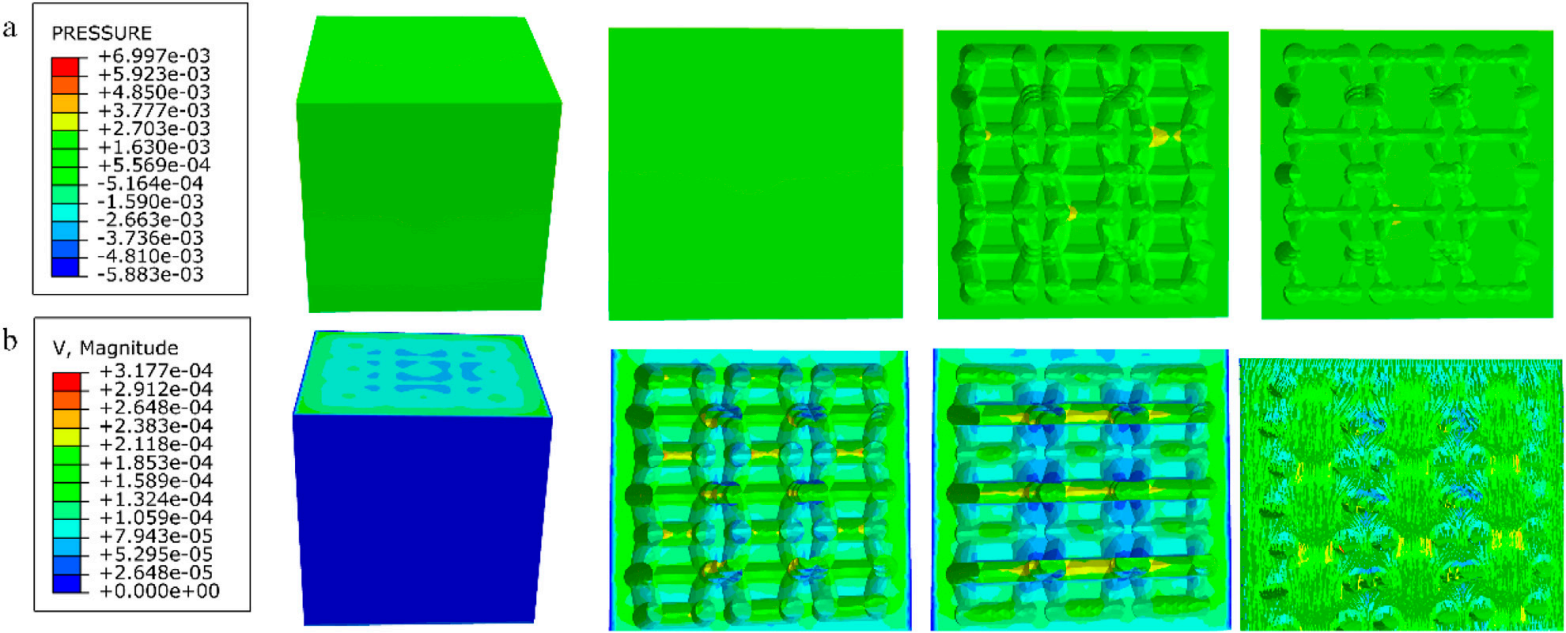
**(a)** Wall shear stress for modified truncated pyramid structure; **(b)** pore flow rate for modified truncated pyramid structure.

The results of the permeability analysis of the four porous structures are shown in Figures 11–14, and the permeabilities of the four porous structures are summarized in Table 4.

**TABLE 4:** Summary of experimental results on permeability of four porous structures.

Parameters\structures	Cubic	Diagonally orientated	Honeycomb	Modified truncated pyramid
Length in the direction of Fluid movement (m)	3.6 × 10^−3^	2.3 × 10^−3^	2.3 × 10^−3^	3.6 × 10^−3^
Peak flow rate (m/s)	2.609 × 10^−4^	2.722 × 10^−4^	3.162 × 10^−4^	3.177 × 10^−4^
Peak pressure drop (Pa)	8.378 × 10^−4^	2.393 × 10^−3^	8.292 × 10^−4^	6.997 × 10^−3^
Permeability (m^2^)	6.231 × 10^−7^	1.636 × 10^−7^	3.672 × 10^−7^	7.46 × 10^−8^

Among them, the permeability of the cubic structure was the highest, 6.231 × 10^−7^ m^2^, and the permeability of the modified truncated pyramid structure was the lowest, 7.46 × 10^−8^ m^2^; the peak velocity of fluid flow between pores was the largest for the modified truncated pyramid structure, 3.177 × 10^−4^ m/s, and the smallest for the cubic structure, 2.609 × 10^−4^ m/s; and the peak pressure drop was the highest for the modified truncated pyramid structure was the highest at 6.997 × 10^−3^ Pa and the lowest at 8.292 × 10^−4^ Pa for the honeycomb type.

### 3.4 Results of a study of degradable porous internal fixation in the treatment of femoral neck fractures

1. Finite element results for internal fixation of 10 mm porous segments

**FIGURE 15:**
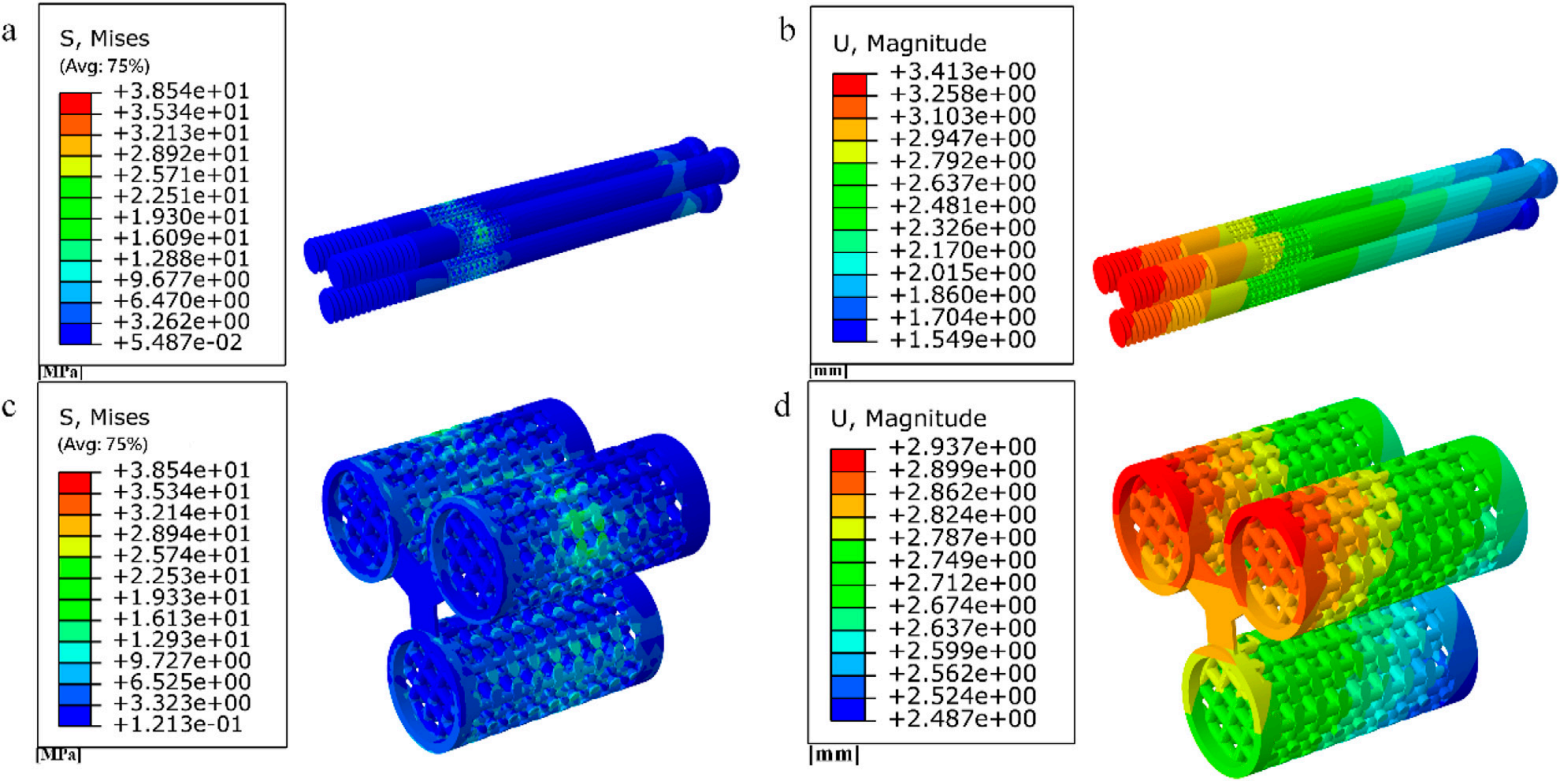
**(a–b)** Stress and displacement distribution of 10 mm cube porous Internal fixation; **(c–d)** Stress and displacement distribution of cube porous structure.

**FIGURE 16:**
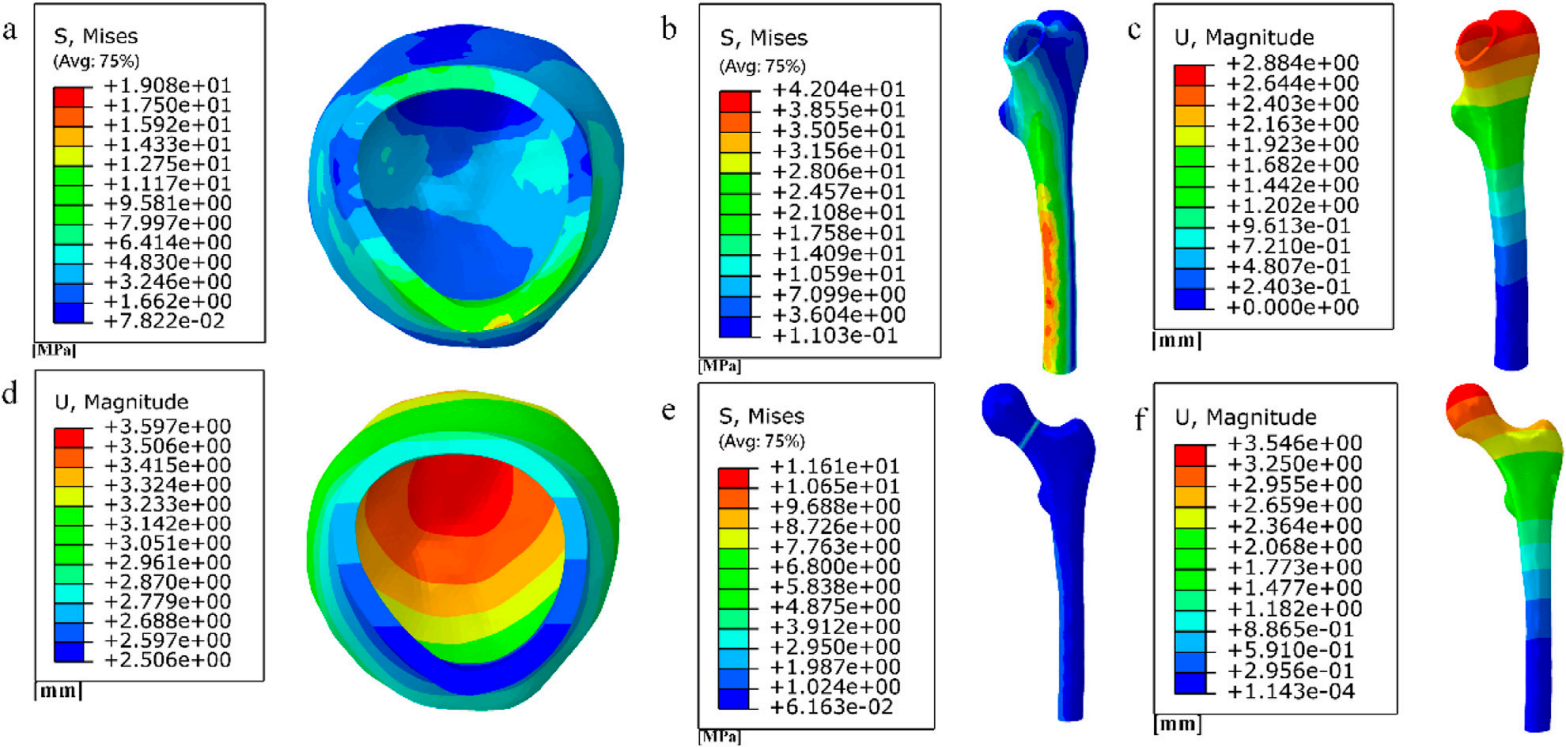
**(a) (b) (e)** Distribution of femoral stresses with 10 mm cube porous internal fixation model; **(c) (d) (f)** Distribution of femoral displacement with 10 mm cube porous internal fixation model.

**FIGURE 17:**
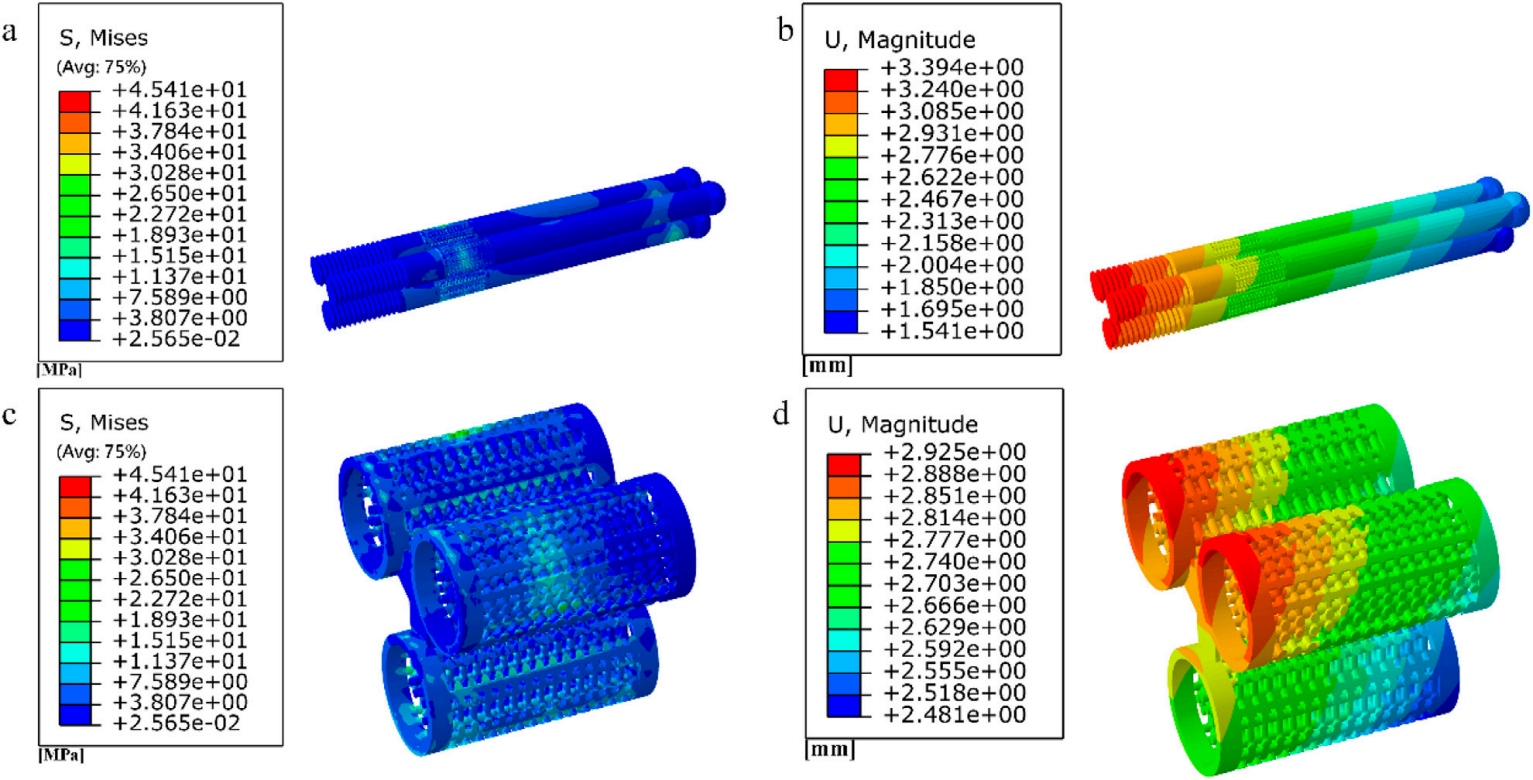
**(a–b)** Stress and displacement distribution of 10 mm honeycomb porous Internal fixation; **(c–d)** Stress and displacement distribution of 10 mm honeycomb porous structure.

**FIGURE 18:**
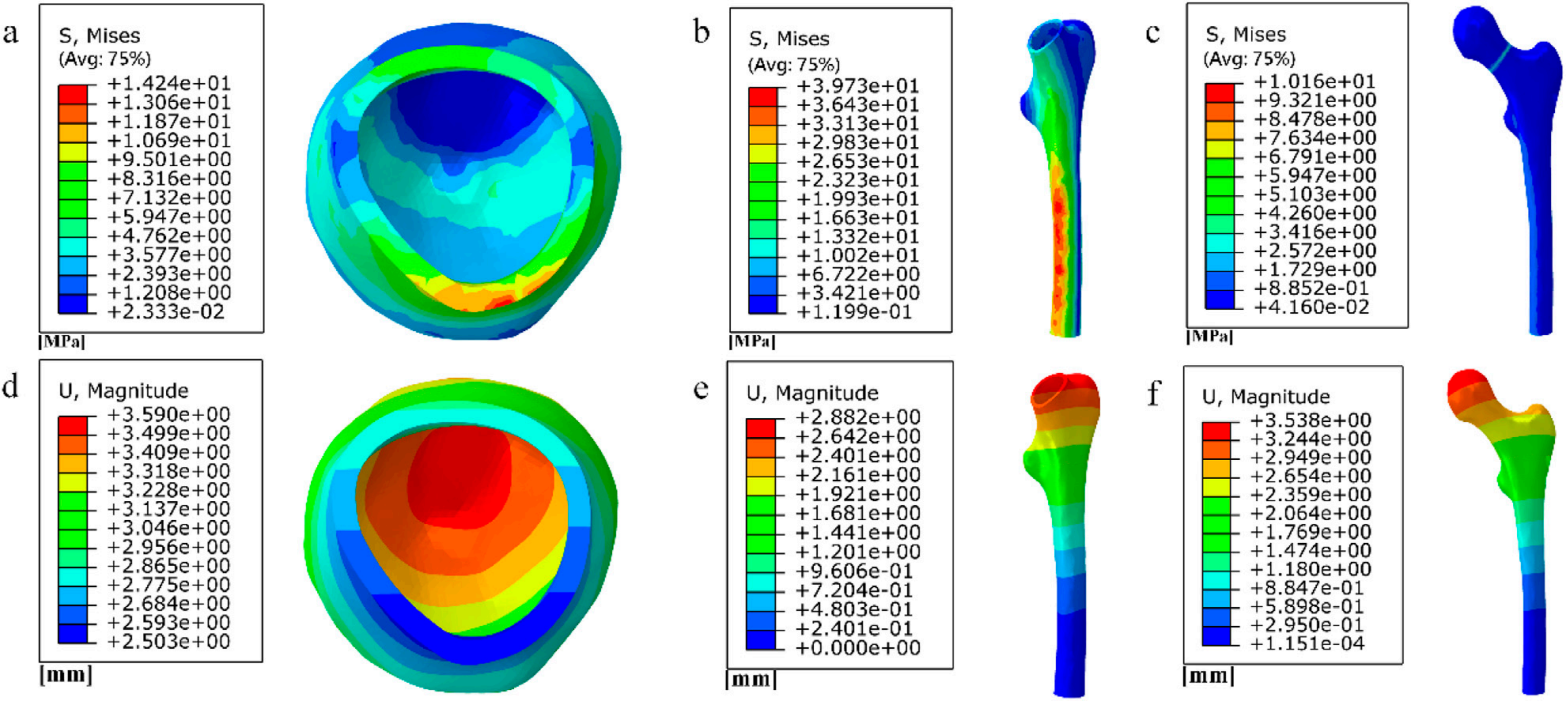
**(a–c)** Distribution of femoral stresses with 10 mm honeycomb porous internal fixation model; **(d–f)** Distribution of femoral displacement with 10 mm honeycomb porous internal fixation model.

The finite element results of internal fixation of 10 mm cube-type porous segments implanted into the fractured femur are shown in Figures 15, 16; the finite element results of internal fixation of 10 mm honeycomb-type porous segments implanted into the fractured femur are shown in Figures 17, 18.

Among them, the peak stress of internal fixation was 38.54 MPa, and the peak displacement was 3.413 mm; the peak stress of proximal fracture block of cortical bone was 19.08 MPa, and the peak displacement was 3.597 mm; the peak stress of distal cortical bone was 42.04 MPa, and the peak displacement was 2.884 mm; and the peak stress of cancellous bone was 11.61 MPa, and the peak displacement was 3.546 mm.

Among them, the peak stress of internal fixation was 45.41 MPa, and the peak displacement was 3.394 mm; the peak stress of proximal fracture block of cortical bone was 14.24 MPa, and the peak displacement was 3.59 mm; the peak stress of distal cortical bone was 39.73 MPa, and the peak displacement was 2.882 mm; and the peak stress of cancellous bone was 10.16 MPa, and the peak displacement was 3.538 mm.

2. Finite element results for full porous internal fixation

**FIGURE 19:**
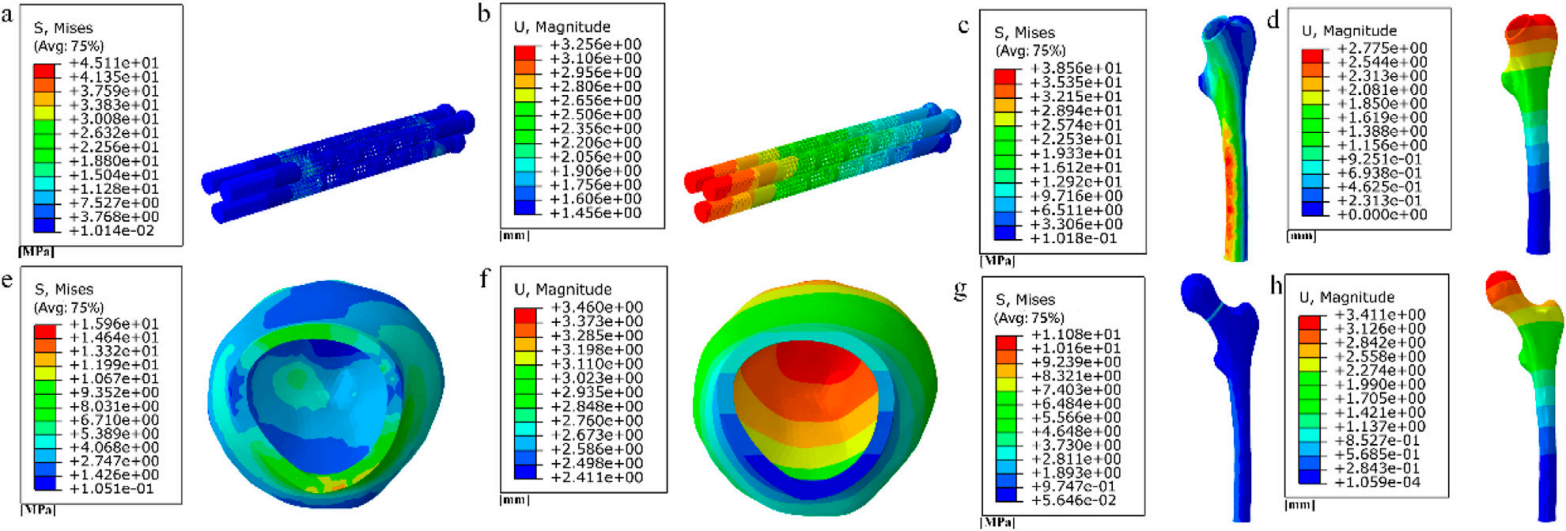
**(a–b)** Stress and displacement distribution on cubic fully porous internal fixation; **(c–h)** Stress and displacement distribution on femur.

**FIGURE 20:**
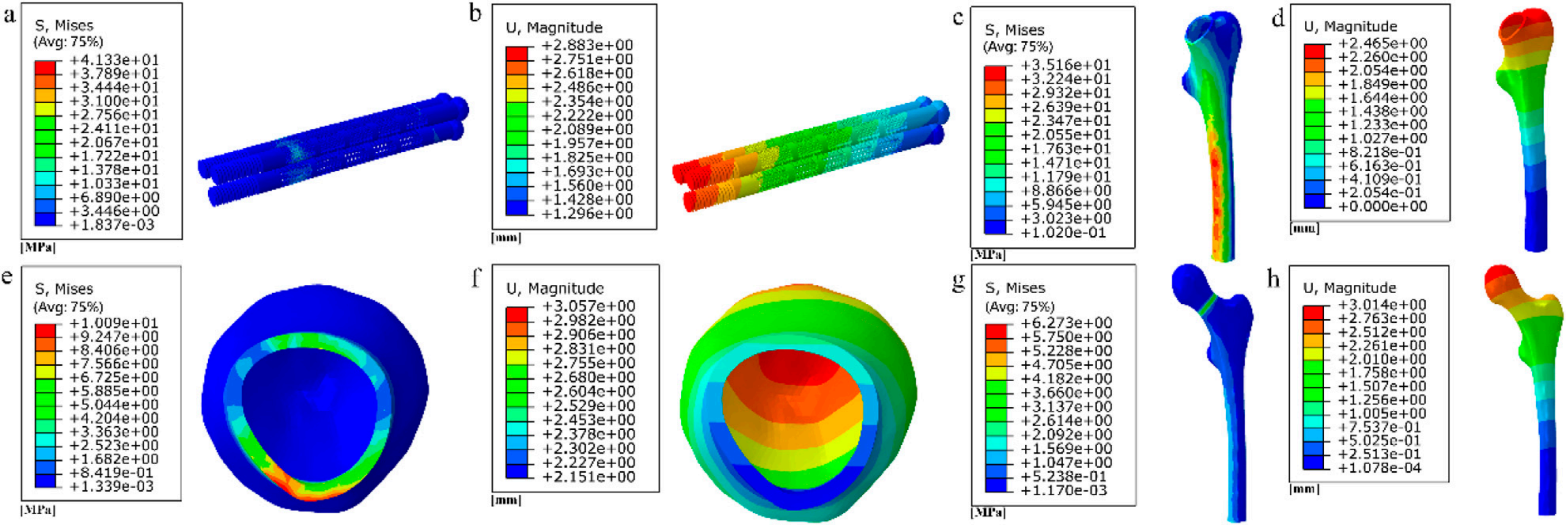
**(a–b)** Stress and displacement distribution on honeycomb fully porous internal fixation; **(c–h)** Stress and displacement distribution on femur.

The finite element results after implantation of the cube-type fully porous internal fixation into the fractured femur are shown in Figure 19, and the finite element results after implantation of the honeycomb-type fully porous internal fixation into the fractured femur are shown in Figure 20.

Among them, the peak stress of internal fixation was 45.11 MPa, and the peak displacement was 3.256 mm; the peak stress of proximal fracture block of cortical bone was 15.96 MPa, and the peak displacement was 3.46 mm; the peak stress of distal cortical bone was 38.56 MPa, and the peak displacement was 2.775 mm; and the peak stress of cancellous bone was 11.08 MPa, and the peak displacement was 3.411 mm.

Among them, the peak stress of internal fixation was 41.33 MPa, and the peak displacement was 2.883 mm; the peak stress of proximal fracture block of cortical bone was 10.09 MPa, and the peak displacement was 3.057 mm; the peak stress of distal cortical bone was 35.16 MPa, and the peak displacement was 2.465 mm; and the peak stress of cancellous bone was 6.273 MPa, and the peak displacement was 3.014 mm.

**FIGURE 21:**
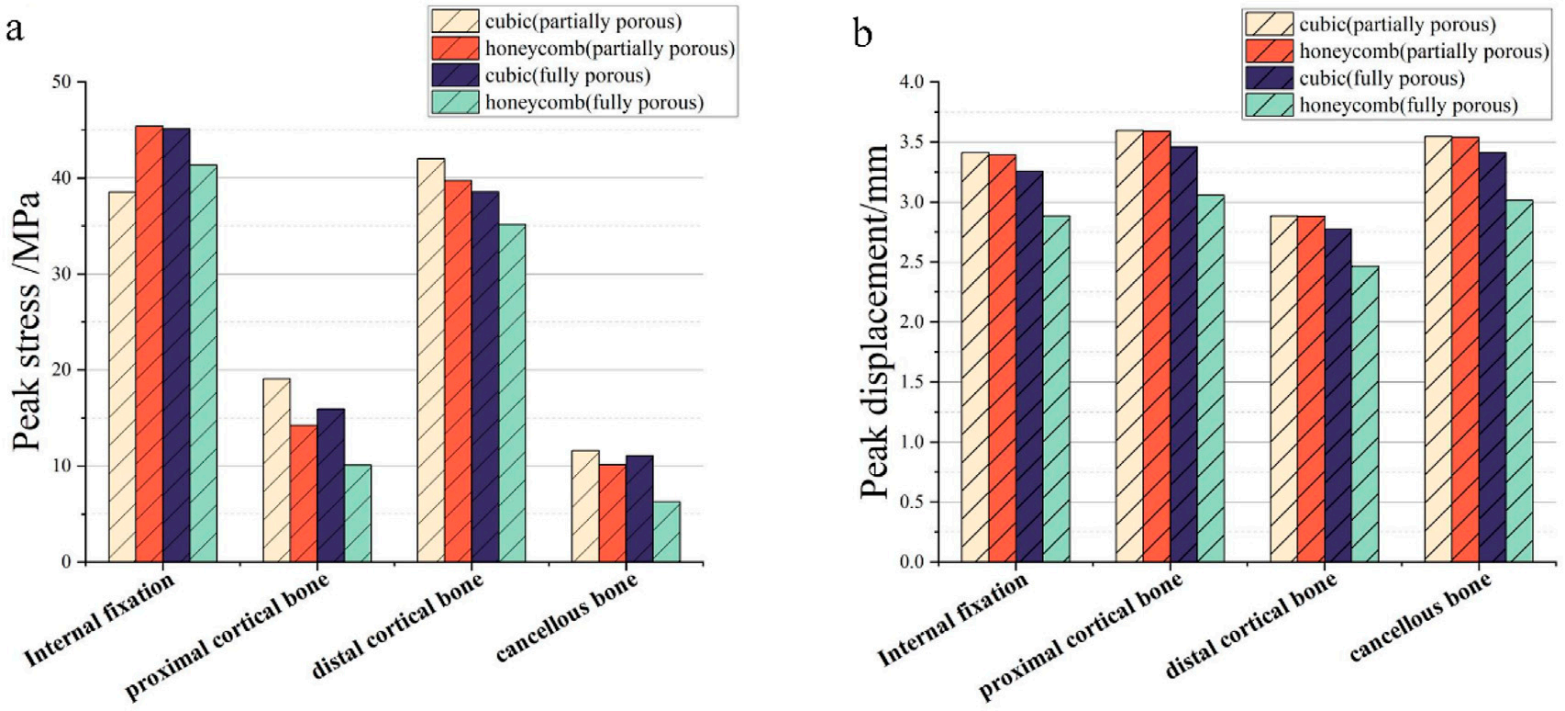
Comparison of peak stress and displacement for porous internal fixation and femur: **(a)** peak stress; **(b)** peak displacement.

The results of the comparison of the two porous structure internal fixation in the partially porous and fully porous state are shown in Figure 21, the honeycomb type structure incorporated into the inverted triangle screw internal fixation, both the 10 mm porous segments and the fully porous, compared with the cube structure internal fixation showed lower peak stresses and peak displacements; internal fixation made fully porous compared to partially porous exhibits lower peak stresses and peak displacements in both honeycomb and cuboidal structures, showing the same pattern as the internal fixation on the femur.

## 4 Discussion

Although sclerotic bone formation along the screw path is recognized as a potential risk factor for avascular necrosis, the proposed two-stage strategy primarily addresses biomechanical optimization in fracture healing. It should be emphasized that this approach currently represents a theoretical framework based on computational evidence. Future validation through animal models and clinical trials is essential to confirm its efficacy in preventing long-term complications.

We have indeed identified a stage where the fracture surface is not yet fully healed, but the femur already possesses sufficient load-bearing capacity, resulting in reduced reliance on internal fixation. At this stage, it is possible to consider early removal of the internal fixation and replacement with a degradable internal fixation, effectively preventing the formation of bone sclerosis. During the healing process, there is minimal disparity in peak displacement on the fracture surface, indicating that the fracture is an instantaneous process of complete rupture, with minimal influence from stress. Stress concentration is observed during the simulation process in regions C and D, and different densities of binding are applied to the non-healed surfaces based on the magnitude of stress in different regions.

With the advancement of 3D printing technology, the precise fabrication of porous structures has become an important support in the field of bone implants. Building on this foundation, the present study focuses on the structural design of a biodegradable AZ31b magnesium alloy porous internal fixation. In contrast to previous studies on porous structures commonly made of titanium alloys (Zhang, 2023; Yang et al., 2023; Peng et al., 2023; Liu et al., 2022), this work specifically aims to meet the requirements of mechanical compatibility and biological transport during fracture healing by designing four types of porous units: cubic, honeycomb, diagonally oriented, and modified truncated pyramid. Methodologically, we combined finite element simulations with computational fluid dynamics analysis to systematically evaluate the equivalent elastic modulus, strength, and permeability of the structures. This approach not only helps reduce stress shielding but also addresses the microenvironmental needs for nutrient transport and bone ingrowth. The integrated analytical method provides a important basis for optimizing the structure of biodegradable porous internal fixation.

The degradation products of magnesium alloys, particularly Mg^2+^ ions, play a crucial role in enhancing osteogenesis and angiogenesis. These ions stimulate bone-forming cells (osteoblasts) and promote new blood vessel formation, which is essential for nutrient supply during healing (An et al., 2025). Furthermore, the gradual degradation of magnesium implants reduces stress shielding over time, allowing natural bone remodeling under physiological loads (Zhao et al., 2016). This article selects AZ31b magnesium alloy with good biocompatibility and designs four porous structural units: cubic, honeycomb, diagonally orientated, modified truncated pyramid. The equivalent elastic modulus and equivalent strength are evaluated through simulated compression experiments to match bone tissue and meet mechanical requirements. The permeability is analyzed using computational fluid dynamics (CFD) to ensure nutrient delivery and bone ingrowth.

The equivalent elastic moduli of four porous structures fall within the range of the elastic modulus of human bone (0.05–20 GPa). Compared to the solid AZ31b material with an elastic modulus of 44.8 GPa, a significant reduction in elastic modulus is achieved. Clinical titanium alloy materials commonly have an elastic modulus of 110 GPa. In contrast, porous structures can effectively reduce stress shielding between implants and human bone. During the validation of the model, the peak stress fixed by the inverted triangular hollow screw group was 36.74 MPa, and the equivalent strengths of the four structures were higher than this value, making them suitable for internal fixation of Pauwels III femoral neck fractures. The distribution of wall shear stress in the fluid domain of the four porous structures is approximately similar. From the inlet to the outlet, the stress decreases. However, when the wall shear force is high, it is not suitable for cell adhesion and growth. The fluid direction near the porous structure follows the structural direction at a certain angle before returning to its original direction. Additionally, the fluid velocity near the porous structure is higher, reaching its peak when the fluid flow vector is tangential to the structure. The permeability lower limit of human bone is 0.5 × 10^−8^ m^2^ (Sun et al., 2023). All four structures meet this condition, but the honeycomb structure has a higher permeability. When it is used for internal fixation in the femur, osteoblasts, osteoclasts, and various nutrients can flow more rapidly between the pores. New bone tissue can adhere and grow more stably on the surface of the structure. As the fracture heals, the internal fixation gradually degrades, and the newly formed bone continues to fill the original position, effectively preventing bone sclerosis. When a 10 mm porous structure is applied for screw fixation in femoral neck fractures, finite element analysis shows that the peak displacement on the femur with honeycomb internal fixation is lower than that with cubic internal fixation. Similarly, the peak stress on the femur is also lower with honeycomb internal fixation compared to cubic internal fixation. When a porous structure is applied to the entire length of the screw, it exhibits the same pattern as the partial porous model. Honeycomb internal fixation demonstrates superior mechanical performance compared to cubic internal fixation. Comparing the partial porous model with the full porous model, under the same structure type, both the internal fixation and the femur in the full porous internal fixation model experience smaller peak stress and displacement, with a more uniform stress distribution and no significant stress concentration.

Although this study demonstrates the potential of biodegradable porous magnesium alloy fixation for improving fracture healing, its findings are primarily based on computational simulations, which may not fully capture *in vivo* biological and mechanical complexities. The degradation behavior of magnesium alloys under realistic physiological conditions and the dynamic interaction between bone ingrowth and implant resorption require further experimental validation. Future work should focus on fabricating and mechanically testing these structures, followed by *in vitro* and *in vivo* studies to assess biodegradation, osteointegration, and long-term biocompatibility, ultimately facilitating clinical translation.

## 5 Conclusion

In conclusion, the use of biodegradable magnesium alloy implants facilitates fracture healing while reducing osteosclerosis risks. By replacing titanium fixation with porous magnesium alloy once cancellous healing is achieved, the implant provides supplementary mechanical support without hindering bone regeneration. The honeycomb-structured design demonstrates superior biomechanical performance, exhibiting more uniform stress distribution and lower peak stress and displacement at the screw-bone interfaces while maintaining sufficient strength to withstand shear forces associated with femoral neck fractures.

## Data Availability

The original contributions presented in the study are included in the article/supplementary material, further inquiries can be directed to the corresponding authors.
